# *EARE-1*, a Transcriptionally Active Ty1/Copia-Like Retrotransposon Has Colonized the Genome of *Excoecaria agallocha* through Horizontal Transfer

**DOI:** 10.3389/fpls.2017.00045

**Published:** 2017-01-24

**Authors:** Jianhua Huang, Yushuai Wang, Wenwen Liu, Xu Shen, Qiang Fan, Shuguang Jian, Tian Tang

**Affiliations:** ^1^State Key Laboratory of Biocontrol, Guangdong Key Laboratory of Plant Resources School of Life Sciences, Key Laboratory of Biodiversity Dynamics and Conservation of Guangdong Higher Education Institutes, Sun Yat-sen UniversityGuangzhou, China; ^2^South China Botanical Garden, Chinese Academy of SciencesGuangzhou, China

**Keywords:** LTR retrotransposon, transcriptional and transpositional activities, horizontal transfer, *Excoecaria agallocha*, euphorbiaceae

## Abstract

Long terminal repeat (LTR) retrotransposons constitute the majority of the content of angiosperm genomes, but their evolutionary dynamics remain poorly understood. Here, we report the isolation and characterization of a putative full-length (~9550 bp) Ty1/*copia*-like retrotransposon in *Excoecaria agallocha* and its evolution in Euphorbiaceae. The so-called *EARE-1* is phylogenetically closely related to *RIRE-1* from *Oryza australiensis*, and has proliferated recently (~7.19 Mya) in the *E. agallocha* genome. An RT-PCR analysis revealed substantial transcription of *EARE-1* in all examined organs (leaves, staminate flowers, pistillate flowers, seeds, and roots) in unstressed *E. agallocha* plants and indications of elevated expression under stress. We conducted sequence analyses of 256 RT-RH fragments (~860 bp) of *EARE-1* from 34 species representing four subfamilies of Euphorbiaceae that exist in China. *EARE-1* copies from two *Excoecaria* species and *Phyllanthus urinaria* showed incongruent phylogeny with the host species and exhibited high sequence similarity to the host genes, suggesting a horizontal transfer from *P. urinaria* to the common ancestor of *Excoecaria*. However, SSAP analysis detected no new insertions of *EARE-1* among full-sibling progeny plants of *E. agallocha*, despite considerable SSAP polymorphisms among half-siblings. *EARE-1* is the first transcriptionally active Ty1/*copia*-like retrotransposon isolated from *E. agallocha*. Our results provide empirical evidence of the horizontal transfer of LTR retrotransposons in plants, and may suggest a significant role of post-transcriptional host control in the life cycles of transposable elements.

## Introduction

Long terminal repeat (LTR) retrotransposons are ubiquitous transposable elements (TEs) that constitute the majority of the content of higher plant genomes (Lee and Kim, 2014). LTR retrotransposons are delimited by LTRs, which contain signals needed for transcription. Autonomous elements contain one open reading frame (ORF) encoding *gag* and *pol* proteins, which are necessary for retrotransposition in a “copy-and-paste” mechanism (Wicker et al., 2007). Following the organization of the *pol* gene, LTR retrotransposons are usually classified into two distinct groups: Ty1/copia and Ty3/gypsy (Kumar and Bennetzen, 1999). Although the dynamics of LTR retrotransposons are critical for the evolution of plant genome size, structure, and function (Liu et al., 2008; Zedek et al., 2010), their own evolution remains poorly understood, especially in non-model organisms with few genomic resources.

The retrotransposon life cycle has been shown to involve multiple steps that are inherently error-prone and mutagenic (Sabot and Schulman, 2006). This leads to numerous remnant elements that are incapable of transposition due to accumulated mutations. Studies have shown that the transposition of retrotransposons is also under strict host control by small RNA-mediated gene silencing at the transcriptional and post-transcriptional levels, which further limits their replication and transmission (Slotkin and Martienssen, 2007; Rigal and Mathieu, 2011). Despite the predominance of inactive elements, it has been reported that bursts of transposition can occur when the host is under genomic shocks such as hybridization, polyploidy, and environmental stresses (Fontdevila, 2005; Casacuberta and González, 2013) or when TEs invade a new “naïve” host genome through horizontal transfer (HT). HT allows TEs to escape host silencing and is thus considered to be an essential step of the TE life cycle, ensuring their long-term survival (Schaack et al., 2010). HT of LTR retrotransposons has been shown to be widespread and frequent in flowering plants (El Baidouri et al., 2014). High sequence similarity between TEs from distantly related taxa, tree incongruence between TEs and the host species and/or patchy distributions of TEs in phylogenies are three lines of evidence commonly used to infer HT (Daniels et al., 1990; Syvanen, 1994; Roulin et al., 2009).

Euphorbiaceae, or the spurge family, is a large, economically important family, with approximately 7500 species organized into 300 genera from five subfamilies: Euphorbioideae, Acalyphoideae, Crotonoideae, Phyllanthoideae, and Oldfieldioideae (Webster, 1994). HT of LTR retrotransposons has been reported in three prominent spurge plants, cassava (*Manihot esculenta*), caster bean (*Ricinus communis*), and Barbados nut (*Jatropha curcas*), for which whole-genome sequences are available (El Baidouri et al., 2014). However, the composition and evolution of TEs remain largely unknown for the vast majority of spurge species, especially those from the subfamily Euphorbioideae, which are characterized by the production of a poisonous milky latex with many medicinal uses (Ernst et al., 2015). Studies on TE dynamics are critical for the understanding of genome evolution and genetic diversity of Euphorbiaceae as a whole.

In this study, we isolated and characterized *EARE-1*, a Ty1/*copia*-like retrotransposon in milky mangrove, or blind-your-eye mangrove (*Excoecaria agallocha*), and surveyed the evolution of *EARE-1* in four subfamilies of Euphorbiaceae that exist in China. Our results suggest that both HT and post-transcriptional control mechanisms of the host may play significant roles in the life cycle of *EARE-1*.

## Materials and methods

### Plant materials and DNA extraction

Leaves, flowers, seeds and roots of *E. agallocha* were collected from Qi Ao Island of Zhuhai City, Guangdong Province, China. Seeds from the same fruit but different individuals of *E. agallocha* were collected and cultivated in a greenhouse until use. Leaves of the Euphorbiaceae species were collected from the Sun Yat-sen University campus and South China Botanical Garden. Voucher specimens were deposited in the Herbarium of Sun Yat-sen University (SYS). DNA was extracted from silica gel-dried leaves using the CTAB method (Doyle and Doyle, 1990).

### Isolation and characterization of *EARE-1*

Partial reverse transcriptase (RT) fragments of Ty1/*copia*-like retrotransposons in *E. agallocha* were amplified using degenerate primer pairs corresponding to the “KTAFLH/NG” and “LLYVDDM/V” conserved motifs (Voytas et al., 1992). PCR amplicons with the expected size (~270 bp) were cloned and sequenced. A total of 32 sequences were acquired. Among them, 25 sequences with a sequence identity of 90–100% were used to design primers for the subsequent inverse PCR. The inverse PCR was conducted as previously described (Syed and Flavell, 2006) except that BamHI, EcoRI, Hind III, or Kpn I (TAKARA) was used for the digestion of the *E. agallocha* genomic DNA. The sequence assembly was conducted using Lasergene (DNASTAR, Inc., Madison, WI, USA), requiring a minimum overlap of 150 bp and a nucleotide identity over 90%. To confirm the sequence assembly, long-range PCR (LA PCR) was conducted using LA Taq (TAKARA) following the manufacturer's instructions. The primers used for LA PCR are listed in Supplementary Table 1. The putative full sequence of *EARE-1* was deposited in GenBank under the accession number KU198316. The structural features of *EARE-1* were identified by manual inspection using BLAST (NCBI).

### Copy number estimation of *EARE-1*

The copy number of *EARE-1* in the *E. agallocha* genome was determined by real-time PCR (qPCR) using the standard curve method described previously (Liu et al., 2016). Primer pairs specific to the LTR, INT, and RT regions of *EARE-1* (Supplementary Table 1) were used for qPCRs using a SYBR® Premix Ex Taq™ kit (TAKARA) on an ABI Prism 7900 HT Real-Time PCR System according to the manufacturer's instructions. DNA samples from three plants were used as three biological replicates. Two technical replicates were run for each sample. Standard curves were established based on a 10-fold dilution series of plasmid DNA, which contained the 5′ LTR of the RT-RH region of *EARE-1*. After adjusting the baseline cycles and calculating threshold values, the number of cycles halfway through the exponential phase (Ct value) was obtained and plotted against the logarithmic value of the standard sample copy numbers to estimate a linear regression line. The relative copy number of *EARE-1* per pg of genomic DNA was then determined based on the linear regression function from the standard curve. The copy number of *EARE-1* was calculated as copies per pg DNA.

### Expression analysis of *EARE-1*

The 5′ and 3′ cDNA ends of *EARE-1* were obtained using a SMARTer® RACE cDNA Amplification Kit (Clontech) according to the manufacturer's instructions. The expression of *EARE-1* in different organs (leaves, staminate flowers, pistillate flowers, seeds, and roots) of unstressed *E. agallocha* plants was determined by reverse transcription PCR (RT-PCR) with three biological replicates. RNA was extracted using a CTAB method as previously described (Yang et al., 2008). The purified total RNA was reverse transcribed into cDNA using the SuperScript® III First-Strand Synthesis System (Invitrogen™) following the manufacturer's instructions. A no-reverse-transcriptase RT reaction was used as the negative control. The PCR was performed using PrimeSTAR® HS DNA Polymerase (TAKARA) with primer pairs specific to the LTR, gag, and RT regions of *EARE-1*. The primers used are shown in Supplementary Table 1.

As for the analysis of stress-responsive expression of *EARE-1*, small pieces (~1 cm^2^) of fresh leaves were immersed in 3 mM MES buffer containing 1 mM salicylic acid (SA), 50 μM 1-naphthylacetic acid (NAA), 200 mM NaCl, or 20% PEG 6000 (drought) in petri dishes at room temperature for 8 h. For the wounding and cold treatment, fresh leaves were narrowly cut vertically, or immersed in MES buffer at 4°C. Total RNA was extracted from treated and untreated (control) leaves, purified, and reverse transcribed as described above. Quantitative real-time RT-PCR (qRT-PCR) of *EARE-1* transcripts was performed using a SYBR® Premix Ex Taq™ kit (TAKARA) with primers specific to the RT region of *EARE-1* (Supplementary Table 1). The relative levels of *EARE-1* were calculated using the 2^∧−ΔΔCT^ method (Livak and Schmittgen, 2001). β*-actin* was used as an internal control. A two-tailed *t*-test was used to determine the significance of the differences in *EARE-1* expression between treated and untreated samples.

### Sequence and phylogenetic analyses of *EARE-1*

Multiple alignments of the RT amino acid sequences of *EARE-1* and known retrotransposons were generated by MUSCLE (Edgar, 2004). The optimal amino acid substitution model was calculated by the ModelGenerator v0.85 (Keane et al., 2006), and the “LG+G” model was selected to construct a maximum-likelihood (ML) phylogenetic tree using PhyML 3.0 software (Guindon et al., 2010) with 1000 bootstrap replicates. *TY3B* from the *gypsy* superfamily was used as an outgroup. The retrotransposon sequences used in the phylogenetic analysis included: *Tork4* (EU105455.1), *Rider* (ABO36622.1), *Ta1-3* (X13291), *Tnt1-94* (X13777), *Tto1* (D83003), *Sto-4* (AF082133), *SORE-1* (AB370254), *RIRE1* (D85597), *BARE-1* (Z17327), *Angela* (AY485644.1), *maximus* (TREP1654), *SIRE1-4* (AY205608.1), *HORPIA* (AY6615581), *leojyg* (AY268139.1), *Tgmr* (U96748), *Retrofit* (AH005614), *TY1B* (Z35766.1), *Bianca* (AF521177.1), and *TY3B* (CAA97115.1).

### Distribution and sequence analyses of *EARE-1* in euphorbiaceae

The PCR amplification of the RT-RH fragments of *EARE-1* and the host gene *rbcL* in the Euphorbiaceae species was conducted using PrimeSTAR® HS DNA Polymerase (TAKARA) with primers listed in Supplementary Table 1. The PCR products were visualized on a 1.8% agarose gel, cloned, and sequenced. The sequences obtained in this study were deposited in GenBank under the accession numbers KU242753–KU243006 for the RT-RH fragments of *EARE-1* and KU243007–KU243040 for *rbcL*. Additional *matK* sequences were retrieved from GenBank: *E. agallocha* (KM255088.1), *E. cochinchinensis* (AB233781.1), *S. discolor* (HQ415366.1), *P. urinaria* (JX661958.1), *M. esculenta* (AB233776.1), *R. communis* (AB233767.1), and *Pedilanthus tithymaloides* (AB268063.1). The sequences of each gene were aligned by codon using MUSCLE (Edgar, 2004) with a manual check. The ML tree was constructed using Fastree (Price et al., 2009) with default parameters and displayed with Figtree v1.4.2. The number of synonymous substitutions per synonymous site (Ks) and the number of nonsynonymous substitutions per nonsynonymous site (Ka) were calculated using the Nei and Gojobori model (Nei and Gojobori, 1986) as implemented in MEGA 6 (Tamura et al., 2013). Alignment gaps were deleted and stop codons were treated as missing data. The codon bias index (CBI) was calculated by DNAsp v5 (Librado and Rozas, 2009).

### SSAP analysis

The SSAP analysis was performed as previously described (Syed and Flavell, 2006; Liu et al., 2016) with some modifications. Briefly, genomic DNA was digested with MseI and EcoRI (NEB) and ligated with adaptors using T4 DNA ligase (NEB). The adaptor-ligated DNA was then used for pre-amplification with primers with no selective nucleotides (E0 and M0) using Ex Taq polymerase (TAKARA). The pre-amplification product was diluted 50-fold for the selective amplification. The fluorescent PCR for selective amplification was performed with primers containing three selective nucleotides and a specific primer (Supplementary Table 2) labeled with FAM. The PCR products of the selective amplification were resolved by capillary electrophoresis and analyzed using Genemarker (Softgenetics, State College, PA).

## Results

### Isolation and characterization of *EARE-1* in *E. agallocha*

**Figure 1:**
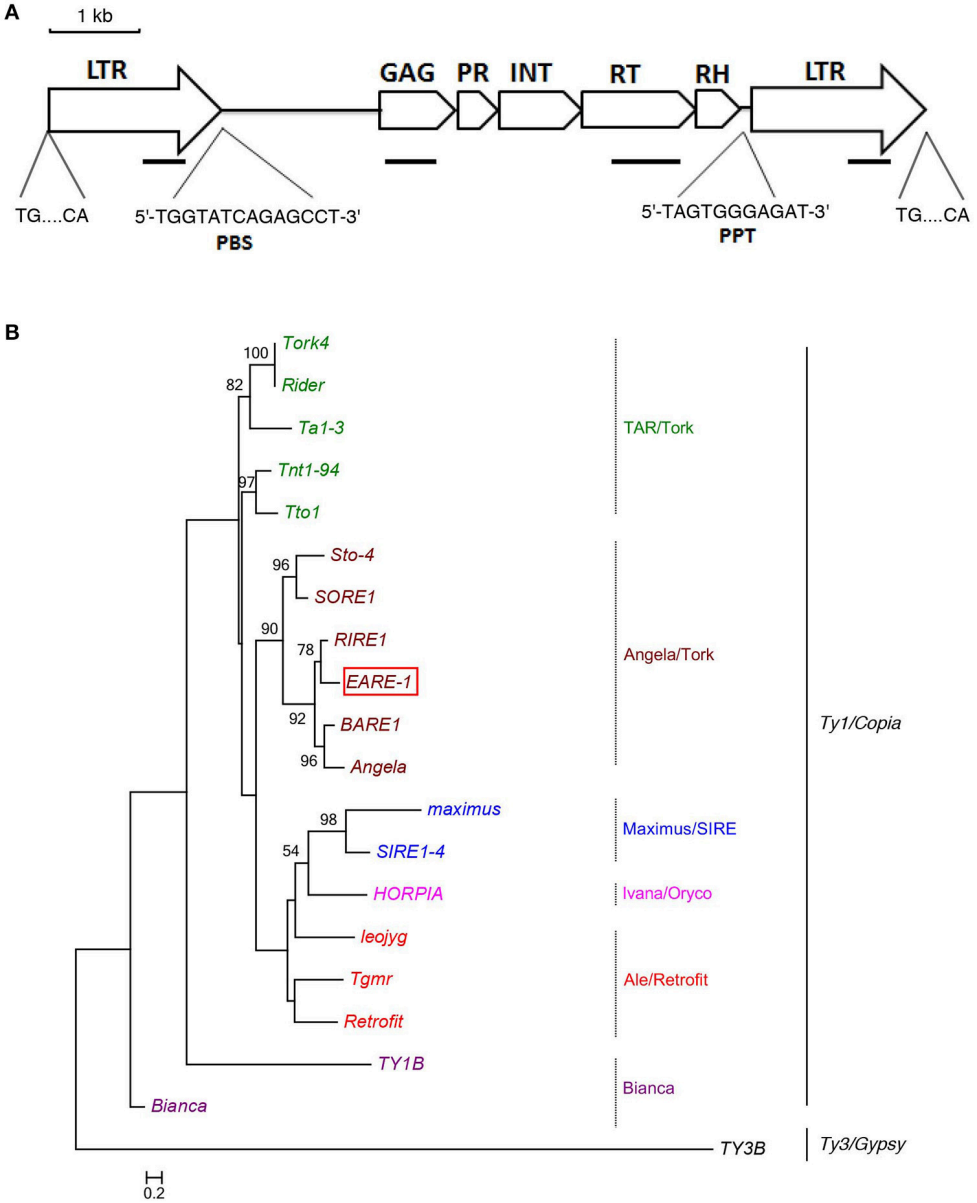
**Structural characteristics of ***EARE-1*** from ***E. agallocha***. (A)** Schematic presentation of *EARE-1*. LTRs and coding regions are indicated with arrows and boxes, respectively. The sequences of PBS and PPT are underlined. Lines indicate the locations of PCR products that were used to estimate the copy number of *EARE-1* in *E. agallocha*. **(B)** ML tree of *EARE-1* and known LTR retrotransposons based on the RT amino acid sequences. Numbers above branches indicate bootstrap values >50% based on 1000 replicates. The retrotransposon sequences used were: *EARE-1* (GenBank accession number: KU198316), *Tork4* (EU105455.1), *Rider* (ABO36622.1), *Ta1-3* (X13291), Tnt1-94 (X13777), *Tto1* (D83003), *Sto-4* (AF082133), *SORE-1* (AB370254), *RIRE1* (D85597), *BARE-1* (Z17327), *Angela* (AY485644.1), *maximus* (TREP1654), *SIRE1-4* (AY205608.1), *HORPIA* (AY6615581), *leojyg* (AY268139.1), *Tgmr* (U96748), *Retrofit* (AH005614), *TY1B* (Z35766.1), *Bianca* (AF521177.1), and *TY3B* (CAA97115.1).

The isolation of a putative full-length Ty1/*copia*-like retrotransposon in *E. agallocha* was initiated by the amplification of partial RT fragments (Voytas et al., 1992), followed by genome walking and sequence assembly as previously described (Lin et al., 2013). The obtained putative complete retroelement as confirmed by LA PCR was 9555 bp in length and was named *EARE-1* (*E. agallocha* retrotransposable element). *EARE-1* included a single ORF of 3920 bp with one stop codon and displayed the *gal*-*pol* domain order of Ty1/copia-like retroelements: *gag*, protease (PR), integrase (INT), reverse transcriptase (RT), and RNase H (Figure 1A). The 5′ and 3′ LTRs of *EARE-1* were 1900 bp and 1916 bp, respectively, with 90% sequence identity, and both had a 5′-TG…CA-3′ structure (Figure 1A). 5′ RACE determined the transcription initiation site (TSS) to be position 918 bp of the 5′ LTR while 3′ RACE located the 3′ end of the *EARE-1* transcripts to position 690 bp of the 3′ LTR. A putative primer-binding site (PBS) of EARE-1 was located 1 bp downstream of its 5′ LTR and contained a stretch of 5′-TGGTATCAGAGCCT-3′ sequence complementary to the 3′ of tRNA^Met^ (Figure 1A). A putative polypurine tract (PPT), which is required for second-strand DNA synthesis, contained a conserved 5′-TAGTGGGAGAT-3′ sequence just upstream of the 3′ LTR of *EARE-1* (Figure 1A). Using the PlantCARE database (Lescot et al., 2002), about 40 TATA-boxes or CAAT-boxes that are common in promoter and enhancer regions and various *cis*-regulatory motifs involved in light, stress, and phytohormone responsiveness and developmental processes were detected in the 5′ LTR of *EARE-1* (Table 1).

**Table 1:** **Types and numbers of putative ***cis***-regulatory elements in the 5′ LTR of ***EARE-1*****.

**Category**	**Motif**	**Organism**	**Sequence**	**Number**	**Function**
Promoter element	TATA-box	Multiple organisms	TATA/TTTTA/TACAAAA/TAATA/TATAAA/ATATAAT/ATATAT/ATATAA/TATATATA	22	Core promoter element around −30 of transcription start
	CAAT-box	Multiple organisms	CAAT/CAATT/CCCAATTT/CCAAT/TGCCAAC/gGCAAT	17	Common cis-acting element in promoter and enhancer regions
Light responsiveness	3-AF1 binding site	*Solanum tuberosum*	TAAGAGAGGAA	1	Light responsive element
	ACE	*Petroselinum crispum*	GACACGTATG	1	cis-acting element involved in light responsiveness
	MRE	*Petroselinum crispum*	AACCTAA	2	MYB binding site involved in light responsiveness
	Box 4	*Petroselinum crispum*	ATTAAT	10	Part of a conserved DNA module involved in light responsiveness
	G-box	*Solanum tuberosum*	CACATGG	1	cis-acting regulatory element involved in light responsiveness
	GATA-motif	*Solanum tuberosum*	AAGGATAAGG	2	Part of a light responsive element
	ATC-motif	*Spinacia oleracea*	AGTAATCT	2	Part of a conserved DNA module involved in light responsiveness
	Box I	*Pisum sativum*	TTTCAAA	1	Light responsive element
	as-2-box	*Nicotiana tabacum*	GATAatGATG	1	Involved in shoot-specific expression and light responsiveness
Stress responsiveness	HSE	*Brassica oleracea*	AAAAAATTTC	1	cis-acting element involved in heat stress responsiveness
	LTR	*Hordeum vulgare*	CCGAAA	3	cis-acting element involved in low-temperature responsiveness
Phytohormone responsiveness	AuxRR-core	*Nicotiana tabacum*	GGTCCAT	1	cis-acting regulatory element involved in auxin responsiveness
	ERE	*Dianthus caryophyllus*	ATTTCAAA	1	ethylene-responsive element
Development	CAT-box	*Arabidopsis thaliana*	GCCACT	1	cis-acting regulatory element related to meristem expression
	Skn-1_motif	*Oryza sativa*	GTCAT	2	cis-acting regulatory element required for endosperm expression
	Circadian	*Lycopersicon esculentum*	CAANNNNATC	1	cis-acting regulatory element involved in circadian control

### The evolutionary relationship between *EARE-1* and major Ty1/*copia* lineages

To identify the evolutionary relationship between *EARE-1* and the known *copia* retrotransposons, we constructed an ML phylogenetic tree based on their deduced RT amino acid sequences, using *TY3B* from the Ty3/*gypsy* superfamily as an outgroup. As shown in Figure 1B, *EARE-1* was most similar to *RIRE-1* from the wild rice *Oryza australiensis* (Noma et al., 1997) and also similar (slightly less so) to the clade of *BARE-1* (Manninen and Schulman, 1993) and *Angela* (Yan et al., 2004). This clustering is supported with high bootstrap values, placing *EARE-1* in the Angela/Tork superfamily (Figure 1B). A homology matrix comparison detected high similarity between the deduced amino acid sequences of *EARE-1* and *RIRE-1*: 45, 49, 70, 68, and 68% for gag, PR, IN, RT, and RNase H, respectively, with an overall identity of 61% across 1317 amino acids. Therefore, *EARE-1* is closely related to *RIRE-1*, which is present in *O. australiensis* in an extraordinary number of copies (Noma et al., 1997).

### Low ratio of LTR to internal region of *EARE-1* in *E. agallocha*

**Figure 2:**
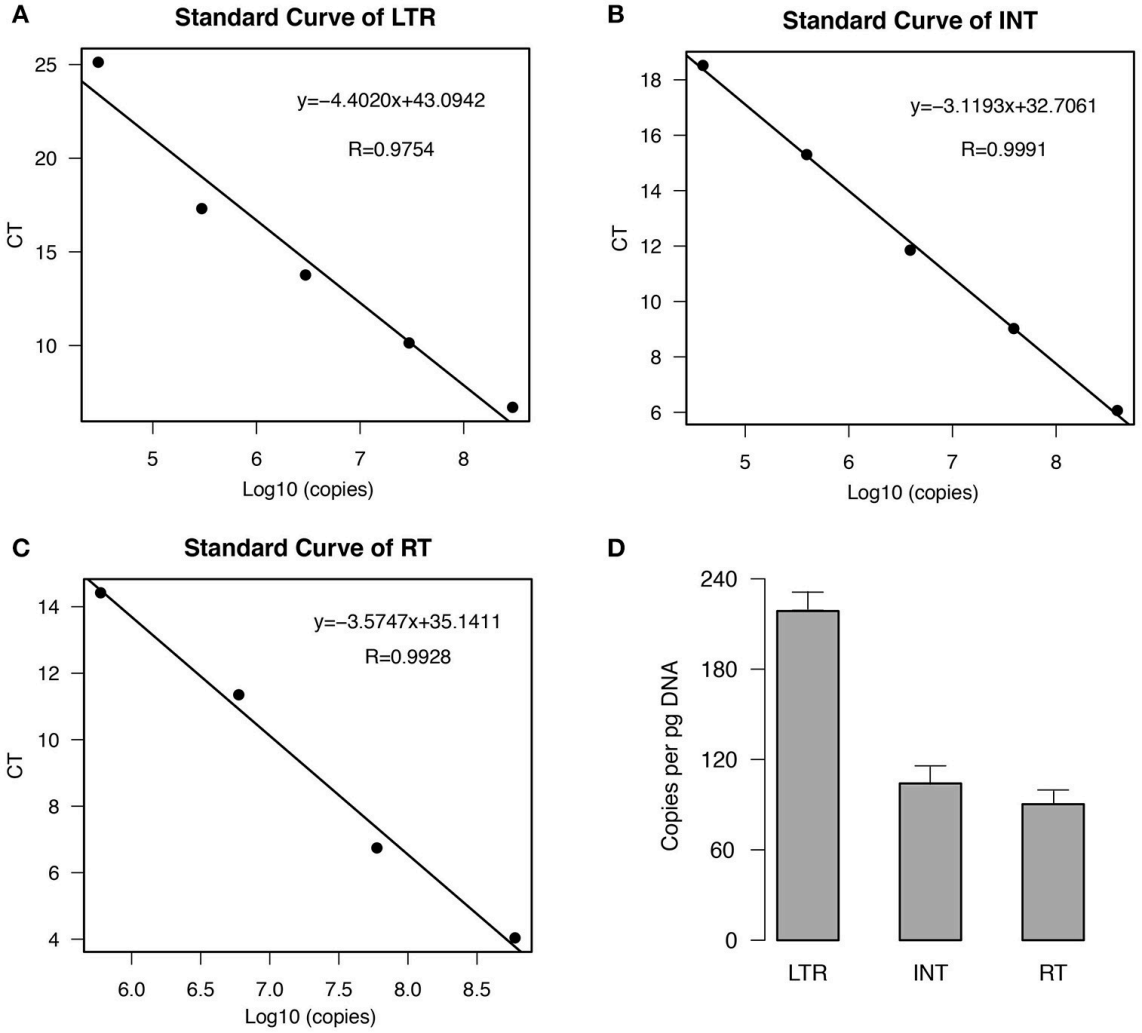
**Estimation of the copy number of ***EARE-1*** in ***E. agallocha*** by qPCR. (A–C)** qPCR standard curves from a serial dilution of plasmid DNAs harboring the cloned *EARE-1* and amplification with LTR/INT/RT primers. The CT values were plotted against the logarithmic value of the standard sample copy numbers. The linear regression function and the correlation coefficient (R) are given for each standard curve. **(D)** Estimation of the *EARE-1* copy number based on the LTR, INT, and RT regions. Error bars indicate the standard deviation (*n* = 3).

To estimate the copy number of *EARE-1* in the *E. agallocha* genome, we conducted qPCR analyses using primers specific to the partial fragments of LTR, IN, and RT. The standard curves, which were generated using a 10-fold dilution series of plasmid DNA containing *EARE-1*, indicated high amplification efficiency for each primer pair (Figures 2A–C). Based on the standard curves, the estimated *EARE-1* copy number per pg DNA was 216 ± 19, 100 ± 14, and 87 ± 12 based on the LTR region and the ORF domains INT and RT, respectively (Figure 2D). The average terminal-to-internal ratio of *EARE-1* was 2.16 (LTR: INT) or 2.48 (LTR: RT), indicating few copies of solo LTRs relative to the full length. These numbers are consistent with, and most simply explained by, the assumption that the majority of copies of *EARE-1* are intact.

### Expression of *EARE-1* in different organs and under stress treatments in *E. agallocha*

**Figure 3:**
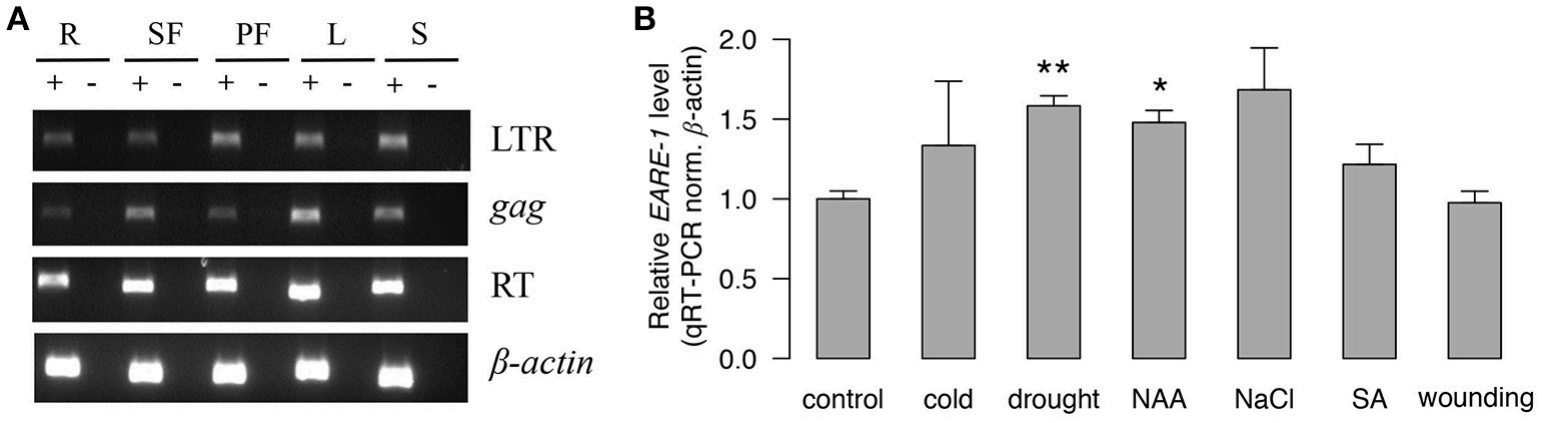
**The transcriptional activity of ***EARE-1*** in ***E. agallocha***. (A)** Expression of *EARE-1* in different organs of *E. agallocha*. R, root; SF, staminate flower; PF, pistillate flower; L, leaf and S, seed. β-actin was used as an internal control. (+): reactions with reverse transcriptase; (−): reactions without reverse transcriptase. **(B)** Stress-responsive expression of *EARE-1* determined by qRT-PCR. The data shown are the relative expression levels of *EARE-1* in the stress-treated vs. untreated (control) leaves of *E. agallocha* normalized using the expression levels of β*-actin*. Cold, 4°C treatment; drought, 20% PEG 6000; NAA, 50 μM 1-naphthylacetic acid; NaCl, 200 mM NaCl; SA, 1 mM salicylic acid and wounding. Three biological replicates were conducted for each treatment. The significance determined by a two-tailed *t*-test is shown as ^*^, *P* < 0.05; ^**^, *P* < 0.01.

The abundant *cis*-regulatory elements found in the LTRs (Table 1) suggested that *EARE-1* may be transcriptionally active and responsive to different internal and external stimuli. Indeed, we found that *EARE-1* was constitutively and ubiquitously expressed at a considerable level in roots, staminate flowers, pistillate flowers, leaves, and seeds of *E. agallocha* using RT-PCR. All three primers specific for the LTR, gag and RT regions amplified amplicons with the expected size (Figure 3A), and these were further confirmed by cloning sequencing. The sequence similarity ranged from 94 to 98% for particular primer pairs, indicating a diverse pool of *EARE-1* transcripts in *E. agallocha*. Using qRT-PCR, we further examined the expression of *EARE-1* in leaves of *E. agallocha* under various stress treatments, including 50 μM 1-naphthaleneacetic acid (NAA), 1 mM salicylic acid (SA), 200 mM NaCl, 20% PEG6000, wounding and cold (see Materials and Methods). *EARE-1* was slightly upregulated in all stress treatments except for wounding, whereas the upregulation was significant only for drought (two-tailed *t*-test, *P* < 0.01) and NAA due to considerable expression variation (two-tailed *t*-test, *P* < 0.05, Figure 3B). These data may indicate constitution expression of *EARE-1* in *E. agallocha* and its elevated expression under stress. However, one needs to be cautious about the stress responsiveness of *EARE-1* as only one internal gene β*-actin* was used in the qRT-PCR analysis.

### The distribution of *EARE-1* in euphorbiaceae and phylogenetic incongruence between *EARE-1* and host species

To understand the evolutionary history of *EARE-1*, we sequenced a partial RT-RNaseH fragment (~860 bp) of the *EARE-1* homologs in 34 species from 31 genera of Euphorbiaceae that represent the four subfamilies that exist in China (Table 2). Amplicons with the expected size were successfully obtained in 27 out of 34 species. Eight to ten clones were randomly chosen and sequenced for each amplicon, resulting in a total of 256 sequences. Two sequences with nucleotide identity <50% with *EARE-1* from *E. agallocha* were removed from further analyses. Species that failed to produce *EARE-1* homologs mainly came from Phyllanthoideae, with an additional two from Crotonoideae or Acalyphoideae (Table 2). The absence of *EARE-1* in *J. curcas* was confirmed by BLAST screening against the spurge species with whole-genome sequences.

**Table 2:** **Summary of the distribution of ***EARE-1*** in Euphorbiaceae**.

**Subfamily**	**Species**	**Voucher**	**PCR ^a^**	**No. of Sequences**
Euphorbioideae	*Euphorbia pulcherrima*	Huang 131101	+	9
	*Excoecaria agallocha*	Huang 131102	+	10
	*Excoecaria cochinchinensis*	Huang 131103	+	9
	*Pedilanthus tithymaloides*	Huang 131104	+	9
	*Sapium discolor*	Huang 131201	+	10
Acalyphoideae	*Acalypha wilkesiana*	Huang 131105	+	10
	*Alchornea trewioides*	Huang 131106	+	9
	*Cephalomappa sinensis*	Huang 131107	+	9
	*Claoxylon indicum*	Huang 131202	+	10
	*Cleidiocarpon cavaleriei*	Huang 131108	−	NA
	*Macaranga tanarius*	Huang 131109	+	8
	*Mallotus anomalus*	Huang 131110	+	10
	*Mallotus apelta*	Huang 131111	+	8
	*Ricinus communis*	Huang 131112	+	10
Crotonoideae	*Aleurites moluccana*	Huang 131113	+	10
	*Codiaeum variegatum*	Huang 131203	+	9
	*Croton tiglium*	Huang 131114	+	10
	*Deutzianthus tonkinensis*	Huang 131115	+	10
	*Jatropha curcas*	Huang 131116	−	NA
	*Manihot esculenta*	Huang 131117	+	10
	*Suregada glomerulata*	Huang 131118	+	10
	*Vernicia montana*	Huang 131119	+	9
Phyllanthoideae	*Baccaurea ramiflora*	Huang 131304	+	9
	*Bischofia polycarpa*	Huang 131120	−	NA
	*Breynia fruticosa*	Huang 131121	−	NA
	*Bridelia tomentosa*	Huang 131122	−	NA
	*Cleistanthus sumatranus*	Huang 131205	−	NA
	*Flueggea virosa*	Huang 131206	+	10
	*Glochidion eriocarpum*	Huang 131123	+	10
	*Phyllanthodendron anthopotamicum*	Huang 131124	+	10
	*Phyllanthus urinaria*	Huang 131207	+	10
	*Richeriella gracilis*	Huang 131125	+	10
	*Sauropus androgynus*	Huang 131126	−	NA
	*Sauropus spatulifolius*	Huang 131208	+	8 (6)^b^

a PCR with bands of expected size is indicated by “+”; the others are indicated by “−.”

b *Sequences with nucleotide identity <50% with EARE-1 from E. agallocha were removed from further analyses*.

**Figure 4:**
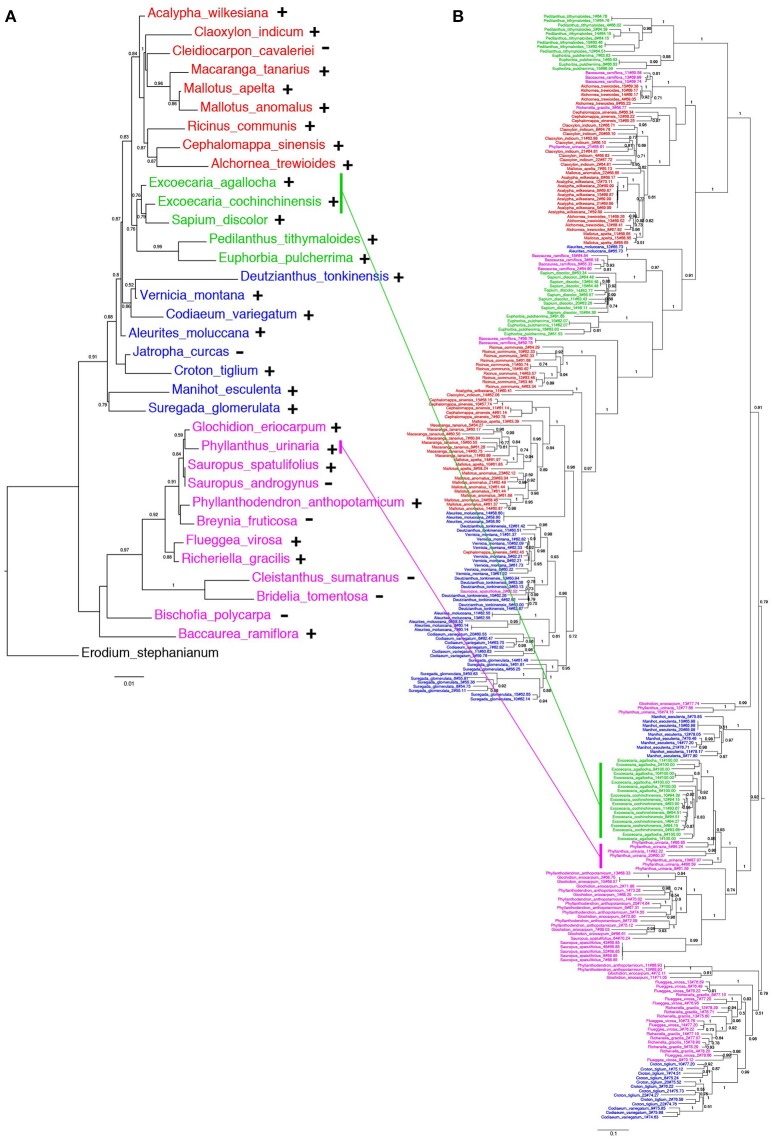
**Comparison of species and ***EARE-1*** phylogenetic histories. (A)** ML tree of the host species Euphorbiaceae based on the partial *rbcL* sequences. The presence or absence of *EARE-1* in each species is labeled with “+” or “−.” **(B)** ML tree of *EARE-1* based on the sequences of the partial RT-RH fragments. The Shimodaira-Hasegawa support values of 0.5 and greater are indicated above the branches. Names of species from the Acalyphoideae, Euphorbioideae, Crotonoideae, Phyllanthoideae subfamilies of Euphorbiaceae are indicated in red, green, blue, and pink, respectively. The sequences of *EARE-1* likely to present a HT event in the gene tree and their corresponding host species in the species tree are connected with straight lines.

The distribution of *EARE-1* in the taxa studied is shown in an ML phylogenetic tree based on host ribulose-1,5-bisphosphate carboxylase/oxygenase (*rbcL*), using *Erodium stephanianum* as an outgroup (Figure 4A). Taxa from Euphorbioideae and Acalyphoideae constituted sister groups and then clustered with those from Crotonoideae, while taxa from Phyllanthoideae diverged distantly from the others. The widespread distribution of *EARE-1* in Euphorbioideae suggests that *EARE-1* entered the common ancestor of Euphorbioideae, was transmitted vertically and underwent independent losses in specific lineages.

In contrast, while individual copies isolated from each of the species mainly formed monophyletic clades in the ML tree of *EARE-1*, the relationships among these clades were not fully congruent with those that connect their host species (Figure 4A vs. Figure 4B). This is most evident when the *EARE-1* sequences from species of distantly related subfamilies of Euphorbiaceae were clustered together (Figure 4B). The phylogenetic incongruence can be explained by either we compared paralogous sequences of *EARE-1*, which were missed in certain lineages due to the limited number of sequenced clones, or HT of *EARE-1* might have occurred between distantly related Euphorbiaceae species.

### High sequence similarity of *EARE-1* between *Excoecaria* species and the divergent *P. urinaria*

**Figure 5:**
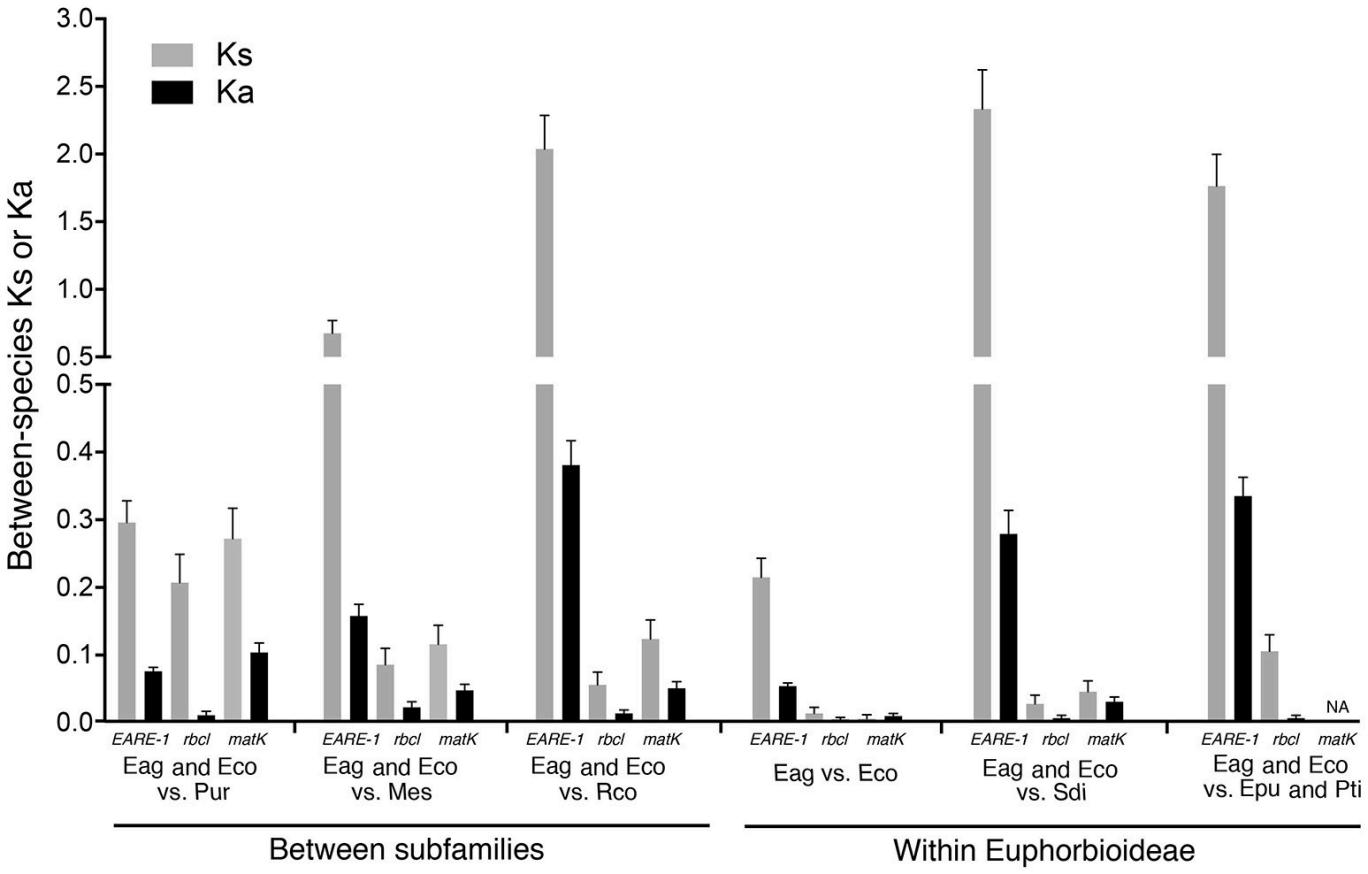
**Mean numbers of substitutions per synonymous site (Ks) and substitutions per nonsynonymous site (Ka) for ***EARE-1*** copies, ***rbcl***, and ***matk*** between ***Excoecaria*** and species from different subfamilies of Euphorbioideae**. Eag, *Excoecaria agallocha*; Eco, *E. cochinchinensis*; Epu, *Euphorbia pulcherrima*; Mes, *Manihot esculenta*; Pti, *Pedilanthus tithymaloides*; Pur, *Phyllanthus urinaria*; Sdi, *Sapium discolor*; Rco, *Ricinus communis*.

As shown in Figure 4, the *EARE-1* sequences from *two Exocaria* species (*E. agallocha* and *E. cochinchinensis*) of Euphorbioideae are surprisingly similar to those from *Phyllanthus urinaria* of Phyllanthoideae. This promoted us to investigate the possibility of HT. HT can be inferred whenever the divergence among TE sequences is significantly lower than that observed for the host genes, assuming similar or higher levels of selective constraints on the latter. To this end, two chloroplast genes, *rbcL* and *matK*, were used for the comparisons of *EARE-1* and host gene divergences between pairs of taxa. The number of synonymous substitutions per synonymous site (Ks) between *Excoecaria* and *P. urinaria* was 0.296 ± 0.032 for *EARE-1*, 0.207 ± 0.042 for *rbcl* and 0.272 ± 0.045 for *matK*, indicating comparable divergence between *EARE-1* and the two host genes (Figure 5). This is quite striking since chloroplast genes are under strong selective constraint, while TEs usually degenerate quickly and thus lack constraint. In contrast, *EARE-1* exhibited a much greater Ks than the host genes when comparing *Excoecaria* with species from other subfamilies of Euphorbiaceae: 6.9–7.9 times greater between *Excoecaria* and *M. esculenta*, Crotonoideae, and 27.2–36.4 times greater between *Excoecaria* and *R. communis*, Acalyphoideae (Figure 5). In addition, the divergence of *EARE-1* between *Excoecaria* and *P. urinaria* was smaller than that between *Excoecaria* and other species within the Euphorbioideae subfamily, while the divergence of host genes showed the opposite trend (Figure 5).

The observed low synonymous divergence of *EARE-1* could be caused by purifying selection for codon usage bias. To rule out this possibility, we calculated the codon bias index (CBI) for both *EARE-1* and host genes in each species of *E. agallocha, E. cochinchinensis*, and *P. urinaria*. A CBI of 0 indicates no codon bias, and a CBI of 1 indicates the most severe codon bias. The CBI values of *EARE-1* (0.33 ± 0.02) were less than that of *rbcL* (*CBI* = 0.45) or *matK* (*CBI* = 0.43), indicating that purifying selection cannot explain the low divergence of *EARE-1* between *Excoecaria* species and *P. urinaria*. Based on these results, we concluded that a horizontal transfer occurred between *P. urinaria* and *Excoecaria* species. The date at which the HT might have occurred can be estimated by calculating the pairwise divergence between all individual *EARE-1* copies from *E. agallocha* and *E. cochinchinensis* and the ancestral founder copy of *P. urinaria* (*Ks* = 0.296 ± 0.032). The result corresponds to an HT occurring at 11.38 Mya based on a molecular clock of 1.3 × 10^−8^ per synonymous substitutions (Ma and Bennetzen, 2004). The small pairwise Ks of *EARE-1* sequences within *Excoecaria* species (*Ks* = 0.187 ± 0.022), corresponding to a burst time of 7.19 Mya, suggest a scenario of recent amplification after the HT.

### The transpositional activity of *EARE-1* in *E. agallocha*

We next looked at whether *EARE-1* is still capable of retrotransposition after its invasion into the *E. agallocha* genome using sequence-specific amplification polymorphism (SSAP) analysis. *E. agallocha* are dioecious trees with three-lobed fruit capsules, where each portion contains a seed. Every three seeds from a fruit produce full-sibling progeny plants, while seeds from different fruits of an *E. agallocha* tree produce half-sibling progeny plants. A total of 153 offspring from 51 fruits of four maternal plants of *E. agallocha* were used for the SSAP analysis. Six primer combinations yielded 268–323 PCR bands for each plant (Supplementary Table 3). Full-sibling plants displayed identical SSAP banding patterns, though half-sibling plants exhibited considerable SSAP polymorphisms, with the percentage of polymorphic bands (P%) ranging from 12.31 to 19.50% (Supplementary Table 3). These results indicate that the transpositional activity of *EARE-1*, if any, is too low to be reliably detected using the current sampling of *E. agallocha*.

## Discussion

We have isolated the first full-length Ty1/copia retrotransposon from the milky mangrove *E. agallocha*. Despite its ancient origin, *EARE-1* is transcriptionally active and has only recently reached hundreds of copies in the genome of *E. agallocha*. Most importantly, phylogenetic incongruence and high sequence similarity provide strong empirical evidence that HT has played a critical role in the evolution of *EARE-1* in *E. agallocha*. The observed low ratio of LTR to the internal region (2.16–2.48) of *EARE-1* indicates either that the efficiency of removal of *EARE-1* by unequal intra-strand homologous recombination (UHR) is low or that a recent burst of transposition has occurred that outpaces DNA removal by recombination. We considered the latter scenario more plausible, since *EARE-1* has long LTRs approximately 1900 bp in length that should be readily subject to UHR.

HT has been shown to preferentially occur in several TE types (Schaack et al., 2010). Consistent with this notion, we found that *EARE-1* is phylogenetically close to *RIRE1*, which is extremely successful (approximately 30,000 complete copies and 10,000 solo-LTRs) in the wild rice *O. australiensis* (Noma et al., 1997; Piegu et al., 2006). After the *RIRE1* burst in *O. australiensis*, multiple HTs of *RIRE1* occurred from *O. australiensis* into other reproductively isolated *Oryza* species (Roulin et al., 2008). Unlike *RIRE1*, the bursts of *EARE-1* in *E. agallocha* and *E. cochinchinensis* probably occurred after the HT from *P. urinaria* into the common ancestor of *Excoecaria*. The estimated HT time of 11.38 Mya, predating the estimated divergence time of *E. agallocha* and *E. cochinchinensis* (5.38 Mya) using a molecular clock of 1.3 × 10^−9^ for *rbcL* (Zurawski and Clegg, 1987), supports this speculation. The fact that the cluster of all horizontally transferred *EARE-1* copies from *E. agallocha* and *E. cochinchinensis* is included in the larger cluster of copies from the Phyllanthoideae subspecies suggests that *E. agallocha* and *E. cochinchinensis* are the recipient species of the HT event. Moreover, the extensive incongruence between the phylogeny of *EARE-1* and that of various host species, such as *Baccaurea ramiflora, Alchornea trewioides, Cephalomappa sinensis*, and *Vernicia montana* (Figure 4), suggests that HTs of *EARE-1* may be frequent within Euphorbiaceae. A comprehensive sampling of more taxa and further sequence analyses of longer or full-length segments of *EARE-1* copies may help to validate this hypothesis.

An active TE copy can experience rapid amplification once it invades into the naive genome through HT. A well-known example is *Rider*, which quickly amplified to approximately 2000 copies after it transferred into the tomato genome from *Arabidopsis* (Cheng et al., 2009; Jiang et al., 2009). In this study, *EARE-1* had reached about 100 copies pre pg DNA in *E. agallocha* (Figure 2) and most of the copies of *EARE-1* are likely to be intact. Considering *E. agallocha* has a large genome (2*n* = 130, Das et al., 2011), the copy number of *EARE-1* in *E. agallocha* would greatly exceeded that in cassava (4 copies) or caster bean (59 copies) estimated by LTR_finder. Interestingly, our results indicate that the life cycle of *EARE-1* is firmly controlled at the post-transcriptional level. This is evident from the contrast between the substantial constitutive expression of *EARE-1* in all analyzed organs of *E. agallocha*—owing to the numerous *cis* promoter and enhancer elements in its LTRs—and its transpositional rate, which was too low to be detected among full-sibling progeny plants. High transcriptional but low transpositional activities were also observed in sunflower hybrid species, in which the proliferation of retrotransposons led to genome expansion (Ungerer et al., 2006; Vukich et al., 2009). Considering the stress-responsive expression of *EARE-1* and the stressful habitats (mangrove swamps) of *E. agallocha*, it would be interesting to study how genomic shock may be associated with HTs in shaping the life cycle of *EARE-1* in *E. agallocha*. A recent study of mangrove retrotransposons may suggest such an association (Liu et al., 2016).

It is unclear how the transfer may have occurred. Considering that *Excoecaria* plants such as *E. agallocha* are well-protected by chemical defenses (Zou et al., 2006), bacteria, fungi, or sapsucking insects that are often believed to be the vectors of HT (Won and Renner, 2003; Fortune et al., 2008; Sun et al., 2013) are less likely to mediate DNA transfer into these species. A possible vehicle may be viruses. In animals, poxviruses, a family of double-stranded DNA viruses, may have acted as vectors for the HT of a SINE element from reptiles to mammals (Piskurek and Okada, 2007). Another group of dsDNA viruses, polydnaviruses (PDVs), are thought to mediate the HT of *mariner*-like elements between a parasitoid braconid wasp and its lepidopteran host (Yoshiyama et al., 2001; Dupuy et al., 2011). *EARE-1* is an attractive candidate for testing whether similar mechanisms of horizontal transposon transfer exist in plants.

In conclusion, *EARE-1* is the first transcriptionally active Ty1/copia-like retrotransposon isolated from *E. agallocha*. Both horizontal transfer and post-transcriptional host control might have played significant roles in the life cycle of *EARE-1*. These mechanisms may be important in understanding the evolution of TEs and TE-driven genomic evolution.

## Author contributions

JH, YW, and TT planned and designed the research; JH, YW, WL, and XS conducted the experiments and data analyses; QF and SJ provided the plant materials; JH, YW, and TT wrote the manuscript. All authors have read and approved the manuscript.

## Funding

This study was funded by the National Science Foundation of China (31170308, 91231117, and 31301010), the Science Foundation for Outstanding Young Teachers in Higher Education of Guangdong (Yq2013005), the Fundamental Research Funds for the Central Universities (16lgjc75) and the Chang Hungta Science Foundation of Sun Yat-sen University.

### Conflict of interest statement

The authors declare that the research was conducted in the absence of any commercial or financial relationships that could be construed as a potential conflict of interest.
