# Supplementary material for: EARE-1, a Transcriptionally Active Ty1/Copia-Like Retrotransposon Has Colonized the Genome of Excoecaria agallocha through Horizontal Transfer
Source: Front Plant Sci. 2017 Jan 24;8:45. doi: 10.3389/fpls.2017.00045 (PMC5258746; doi:10.3389/fpls.2017.00045)
Supplement: Supplementary file 1 [file Table1.DOCX]

**Supplementary Table 1** Primers used in this study.

| Long-range PCR | |
| --- | --- |
| LA1F | TGTTAGTGTAAGCCCTAGCCCCAAT |
| LA1R | AACTCCGGGCCTATGAATGCACC |
| LA2F | TTAGTCCATTGGGCTTTGGCCC |
| LA2R | GAACCTAAGATCAAGTAAATGGCCG |
| LA3F | TTGGATGCACCCTTGCCTGAAG |
| LA3R | CTATGCTCCCACTAACCTTCTT |
| LA4F | AGCAAATGGATGTTAAAACGGCTTTCCTC |
| LA4R | GAGCTTCGTGCATATTTGGACGC |
| LA5F | TCGTGGGGACGTCAAGGTTAGCAAG |
| LA5R | TGATGGAATATGACATGGTATGATAAT |
| Copy number estimation | |
| LTRqF | CGAAATACACAAGGAGAAGGATTC |
| LTRqR | GGTGATGACCAGCAACTAAGA |
| intqF | TCTGGTATTGTCGTTTAGGACAT |
| intqR | CAATAAGCCATTAGTCCTTTCAC |
| RTqF | TACACAAGTACCTAGAAGGTCT |
| RTqR | TCATGGCTTCAAGCCAATTTTC |
| Rapid amplification of cDNA ends |  |
| 5'RACE primer | AACCTTGTGGGCTGAATTTCTCTCT |
| 3'RACE primer | TGTTAGTGTAAGCCCTAGCCCCAAT |
| Expression analyses | |
| LTRF | GGAATTTCGGCACATGCTAT |
| LTRR | GATCACCAATGCCAAGGAAC |
| gagF | CTGCTAGGAACGCTTATGTGG |
| gagR | CGGGTCCCTTCTTAATTCCCTTT |
| RTF | CGAGACCTTTTCGCCTGTAG |
| RTR | CTGACCAGTGCTTCTCACCA |
| actinF | GGGAAATTGTCCGTGACATGAA |
| actinR | CCTTGGAAATCCACATCTGC |
| Distribution analyses of *EARE-1* in Euphorbiaceae | |
| RT-RH-F | TGTGTACAAGAAGGTTAGTGGGAG |
| RT-RH-R | ATGTGTTTGGATTTCTGGTGTG |
| *rbcL*-F | ATGTCACCACAAACAGAAAC |
| *rbcL*-R | CATGTACCTGCAGTAGC |
