# Supplementary material for: EARE-1, a Transcriptionally Active Ty1/Copia-Like Retrotransposon Has Colonized the Genome of Excoecaria agallocha through Horizontal Transfer
Source: Front Plant Sci. 2017 Jan 24;8:45. doi: 10.3389/fpls.2017.00045 (PMC5258746; doi:10.3389/fpls.2017.00045)
Supplement: Supplementary file 2 [file Table2.DOCX]

**Supplementary Table 3** SSAP analysis of *EARE-1* polymorphisms in 153 offspring of *E. agallocha* from four individuals. n, number of polymorphic bands; P%, percentage of polymorphic bands.

| Primers | Individual 1  (Fruit = 11) | | |  | Individual 2  (Fruit = 11) | | |  | Individual 3  (Fruit = 5) | | |  | Individual 4  (Fruit = 24) | | |
| --- | --- | --- | --- | --- | --- | --- | --- | --- | --- | --- | --- | --- | --- | --- | --- |
|  | Total  bands | Polymorphic  bands | |  | Total  bands | Polymorphic  bands | |  | Total  bands | Polymorphic  bands | |  | Total  bands | Polymorphic  bands | |
|  |  | n | P% |  |  | n | P% |  |  | n | P% |  |  | n | P% |
| E2  E3  E4  E5  E6  E7  Total | 94  60  32  48  47  42  323 | 18  8  6  13  9  9  63 | 19.14  13.33  18.75  27.08  19.15  21.42  19.50 |  | 84  61  29  43  44  43  304 | 15  8  6  5  6  7  47 | 17.86  13.11  20.68  11.63  13.64  16.27  15.46 |  | 63  57  29  40  39  40  268 | 9  7  5  5  4  3  33 | 14.29  12.28  17.24  12.50  10.25  10.00  12.31 |  | 101  52  22  41  44  43  303 | 18  10  4  10  6  7  55 | 17.82  19.23  18.18  24.39  13.64  16.27  18.03 |
