# Supplementary material for: EARE-1, a Transcriptionally Active Ty1/Copia-Like Retrotransposon Has Colonized the Genome of Excoecaria agallocha through Horizontal Transfer
Source: Front Plant Sci. 2017 Jan 24;8:45. doi: 10.3389/fpls.2017.00045 (PMC5258746; doi:10.3389/fpls.2017.00045)
Supplement: Supplementary file 3 [file Table3.DOCX]

**Supplementary Table 2** SSAP adaptor and primer sequences

| **Type** | **Name** | **Sequence (5′-3′)** |
| --- | --- | --- |
| Retrotransposon primer | LTR-1 | CATAACAATGACTGACTAGGG |
| Double-strand adaptors | *EcoRI* adaptors | CGTAGACTGCGTACC |
|  |  | AATTGGTACGCAGTCTAC |
|  | *MseI* adaptors | GACGATGAGTCCTGAG |
|  |  | TACTCAGGACTCAT |
| Adaptor-specific primers | E0(*EcoRI*+A) | GACTGCGTACCAATTC |
|  | M0(*MseI*+C) | GATGAGTCCTGAGTAA |
|  | E2(*EcoRI*+AAG) | GACTGCGTACCAATTC AAG |
|  | E3(*EcoRI*+AGC) | GACTGCGTACCAATTC AGC |
|  | E4(*EcoRI*+AAC) | GACTGCGTACCAATTC AAC |
|  | E5(*EcoRI*+ACC) | GACTGCGTACCAATTC ACC |
|  | E6(*EcoRI*+ACT) | GACTGCGTACCAATTC ACT |
|  | E7(*EcoRI*+AAT) | GACTGCGTACCAATTC AAT |
